# Simulated Angiography Using a Bleomycin Mixture for Sclerotherapy of Lymphatic Malformations

**DOI:** 10.3389/fped.2020.563517

**Published:** 2020-09-24

**Authors:** Lei Guo, Changhua Wu, Xiaoyan Li, Dan Song, Jiali Sun, Yunkui Zhang

**Affiliations:** ^1^Department of Vascular Anomalies and Interventional Radiology, Qilu Children's Hospital of Shandong University, Jinan, China; ^2^Department of Ultrasound, Second Affiliated Hospital of Shandong University of Traditional Chinese Medicine, Jinan, China

**Keywords:** sclerotherapy, bleomycin, lymphatic malformations, image-guided, US-DSA-guided

## Abstract

**Objective:** Repeat sclerotherapy of lymphatic malformations (LMs) is challenging. Accordingly, the aim of the present article is to describe a simulated angiography technique—a new method of bleomycin infusion for the treatment of LMs to achieve better outcome(s) in fewer sessions.

**Materials and Methods:** A retrospective analysis of information housed in a prospectively collected LM database was performed. Patients with LM, revealed on imaging examination and treated using a simulated angiography technique with a bleomycin mixture, were included in the study. Visual evaluation and imaging examinations were performed to evaluate clinical improvement.

**Results:** A total of 151 patients (82 male, 69 female; mean age, 28.29 months [range 1 month−12 years]) with LMs were included in this study. Excellent visual and radiological resolution was observed in 77% (117/151) of lesions, 17% (26/151) had significant improvement, and 8 patients exhibited a slight response. The number of procedures per patient varied from 1 to 5, and the average number of treatment sessions for LM was 1.34. Side effects included skin erythema at the injection site, local swelling, mild tenderness, and fever, which were controlled by oral antipyretics. No serious side effects were observed.

**Conclusions:** Simulated angiography using a bleomycin mixture for sclerotherapy of LMs in children was feasible and demonstrated good effect with little trauma and less time per treatment.

## Introduction

Lymphatic malformations (LMs)—traditionally known as lymphangiomas—are benign vascular lesions caused by developmental disorders of the lymphatic system in the embryonic stage. These lesions are most commonly found in the head and neck region, accounting for 45–52% of all cases (1), which have significant propensity for appearing at birth and, even prenatally (2), with 90% clinically apparent at 2 years of age (3). LMs commonly appear as localized areas of swelling associated with viral or bacterial infections, intralesional hemorrhage, inflammation, and/or trauma (4), and consist of abnormally formed lymphatic channels and cystic spaces filled with eosinophilic- and protein-rich fluid (1). Based on the classification of the International Society of the Study of Vascular Anomalies, cystic LMs are divided into three categories: macrocystic, microcystic, and combined. Treatment options for LMs include resection, sclerotherapy, laser coagulation, and radiofrequency ablation, although some of these methods are often combined (5). Surgery is no longer a primary treatment option because it is difficult to perform complete excision without scarring, which is associated with recurrence or regrowth of the LMs (6). Image-guided percutaneous sclerotherapy, using an agent such as bleomycin, is widely used to shrink cystic LMs. However, the use of ultrasound (US) guidance alone is sometimes unable to distinguish the sclerosing agent from the original cyst fluid, which often leads to missed lesions and prolonged treatment time (7). Simulated angiography using a bleomycin mixture based on a combined protocol of US and digital subtraction angiography (DSA) is a novel and evolving treatment for LMs. We describe the bleomycin administration procedure used in our institution for lymphatic malformation (LM) component and the bleomycin safety profile. Preprocedural and postprocedural clinical data and MR imaging were used to objectively and subjectively demonstrate the efficacy of this procedure.

## Materials and Methods

### Pre-procedural Work-Up

The human subject protocol was approved by the Committee on Clinical Investigation of the University. Informed consent was obtained from each patient in accordance with the World Medical Association Declaration of Helsinki. All methods were performed in accordance with relevant guidelines and regulations (http://jama.jamanetwork.com/article.aspx?articleid=1760318). Informed consent for publication of anonymized information/images in an online open-access publication was issued.

Most patients visited the outpatient department presenting with superficial masses or other symptoms, such as pain, inflammation, and compression of adjacent organs, depending on the size of the lesion, anatomical location, and complications. Pre-procedural US was performed for positive diagnosis in the outpatient department. Further unenhanced magnetic resonance imaging (MRI) examination is essential for classification, extent, depth, number, and distribution of the cystic cavity (8). Associated lymphatic vessel abnormalities and, sometimes direct communication(s) between the LM and adjacent lymphatic vessels, are also depicted (9). Pre-procedural MRI also enables the construction of templates for a single-frame imaging under the DSA protocol to avoid missing foci and to determine whether the drug is evenly distributed. Indications for the percutaneous treatment of LMs included swelling, pain, and cosmetic and functional problems. Patients with normal electrocardiogram, blood coagulation, liver function, renal function, and routine blood examination results were included. Individuals with surgical contraindications, however, were excluded. Patients or their parents were informed about possible complications and risks related to percutaneous treatment before the procedure, and written informed consent was obtained from all. Additionally, informed consent for publication of anonymized information/images in an online open-access publication was obtained from the patients' parents or their legal guardians.

### Preparation of the Bleomycin Mixture

The mixture prepared for percutaneous sclerotherapy consists of 15 mg of bleomycin and 7.5 ml of contrast agent mixed in a syringe. Dexamethasone is added during every process to attenuate local responses following the operation and to dilute concentration. The therapeutic dose of bleomycin depends on the type, extent, and size of the lesion, and its relationship with the surrounding tissue to achieve a margin of safety (0.3–0.5 mg/kg, maximum single dose 8 mg).

### Stepwise Approach to Simulated Angiography Using a Bleomycin Mixture

The procedure was performed under general anesthesia and laryngeal mask ventilation in all children. Intralesional sclerotherapy was administered by percutaneous injection through appropriate needles according to the size and depth of the lesion under US guidance. Rotation of needles is useful for breaking through the cyst wall. After the needle reaches its ideal position within the LM, a yellow transparent liquid or light red non-condensable liquid is slowly aspirated from LM under US monitoring through a syringe. The lesions were punctured at 1–3 sites according to their size. Four-to-8 needles inserted into the microcystic component of the lymphatic malformations were used in each session, depending on the volume of mixture available based on the patient's weight. It is important to maintain the original position of the needle(s) depicted on US in following treatments of other foci to avoid omission. To avoid overfilling and leakage, different concentrations of sclerosis agents are selected to be injected according to the volume of cyst fluid. After injection, bandage compression was used so that the solution is evenly distributed over the inner wall of the capsule. Before completion of the operation, a single-frame image is captured under DSA fluoroscopy and compared with MRI to determine whether there are any missing lesions to avoid omission of an unwitting cavity given that the sclerosing agent does not penetrate. In addition, a supplementary injection of bleomycin mixture is required if there is capsular space.

### Follow-up and Assessment

All cases in the present study were followed-up 1 month after the initial treatment, and evaluated by the clinician according to the aim of the treatment: symptom improvement, reduction of the size until it becomes invisible, and to avoid complications. The clinician, who was blinded to the treatment, judged the treatment effect(s) by comparing the pre- and post-treatment images at the same angle. Visual evaluation of improvement of LMs was performed using a scale scored between 0 and 5, in which a score of 0 represented worsening and 1 represented no change, whereas 2 (<25%), 3 (26–50%), 4 (51–75%), and 5 (≥75%) represented, mild, moderate and significant improvement, and excellent resolution, respectively.

At the same time, response to treatment was also evaluated by imaging examination, including US and MRI, by comparing pre- and postoperative cystic volumes. The volume was calculated using the following equation:

$$\begin{array}{l} \text{width}\times \text{depth}\times \text{height}\times \pi /6\left(\text{width}\times \text{depth}\times \text{height}\times 0.52\right) \end{array}$$

This was based on the assumption that the lesions were round or oval. Reduction in lesion volume was estimated by subtracting the post-treatment volume from the pre-treatment volume. The proportion of shrinkage was then estimated by reduced volume divided by pre-treatment volume then multiplying by 100, which was also subsumed into the same 0–5 scale described above. Sclerotherapy sessions were repeated when necessary. If not, they were scheduled in the next cycle of follow-up until a final decision was made regarding the end of treatment or loss to follow up. SPSS version 19.0 for windows was utilized to conduct all statistical analyses. The *t*-test and chi-square test were used for univariate analysis of continuous and categorical data, respectively. Statistical significance was defined by *P* < 0.05.

## Results

A total of 151 patients (82 male, 69 female; mean age, 28.29 months (range 1 month−12 years]) with LMs were included in this study. Demographic and clinical data are summarized in Table 1. The overwhelming majority of patients complained of local masses and cosmetic issues (*n* = 142). Ten patients also experienced pain, and four exhibited functional problems.

**Table 1:** Baseline clinicopathological characteristics of patients with LMs.

**Characteristic**	**Total**		**Classification**					
	***N*** **=** **151**		**Macrocystic**		**Microcystic**		**Combined**	
			***N*** **=** **72**		***N*** **=** **18**		***N*** **=** **61**	
Female/male	69/82		31/41		9/9		29/32	
Average age (M)	28.29		31.53		27.08		24.8	
Number of sessions	1.34		1.26		1.45		1.41	
Total dose (mg)	1.93		2.03		2.20		1.73	
**Chief complaint**								
Local masses	142		68		18		56	
Pain	10		6		0		4	
Dysfunction	4		2		0		2	
**Position**								
Head and neck	83		42		8		33	
Trunk	48		23		5		20	
Limb	31		14		7		10	
Outcome	R	V	R	V	R	V	R	V
0	0	0	0	0	0	0	0	0
1	0	0	0	0	0	0	0	0
2	2	1	0	0	1	1	1	0
3	6	8	0	1	3	3	3	4
4	26	25	3	2	5	5	18	18
5	117	117	69	69	9	9	39	39

*R, radiological evaluation; V, visual evaluation*.

Lesions were located in the head and neck region in 83 patients, in the trunk in 48, in the limb in 31, and a combination thereof in 8. Aspiration was performed in four patients whose lesions had close proximity to the airways. Seventy-two lesions were macrocystic (>2 cm^3^), 18 were microcystic, and others were combined. Bleomycin was administered as a percutaneous sclerotherapy agent to all patients. Sixty of the 151 patients had a history of surgery.

**Figure 1:**
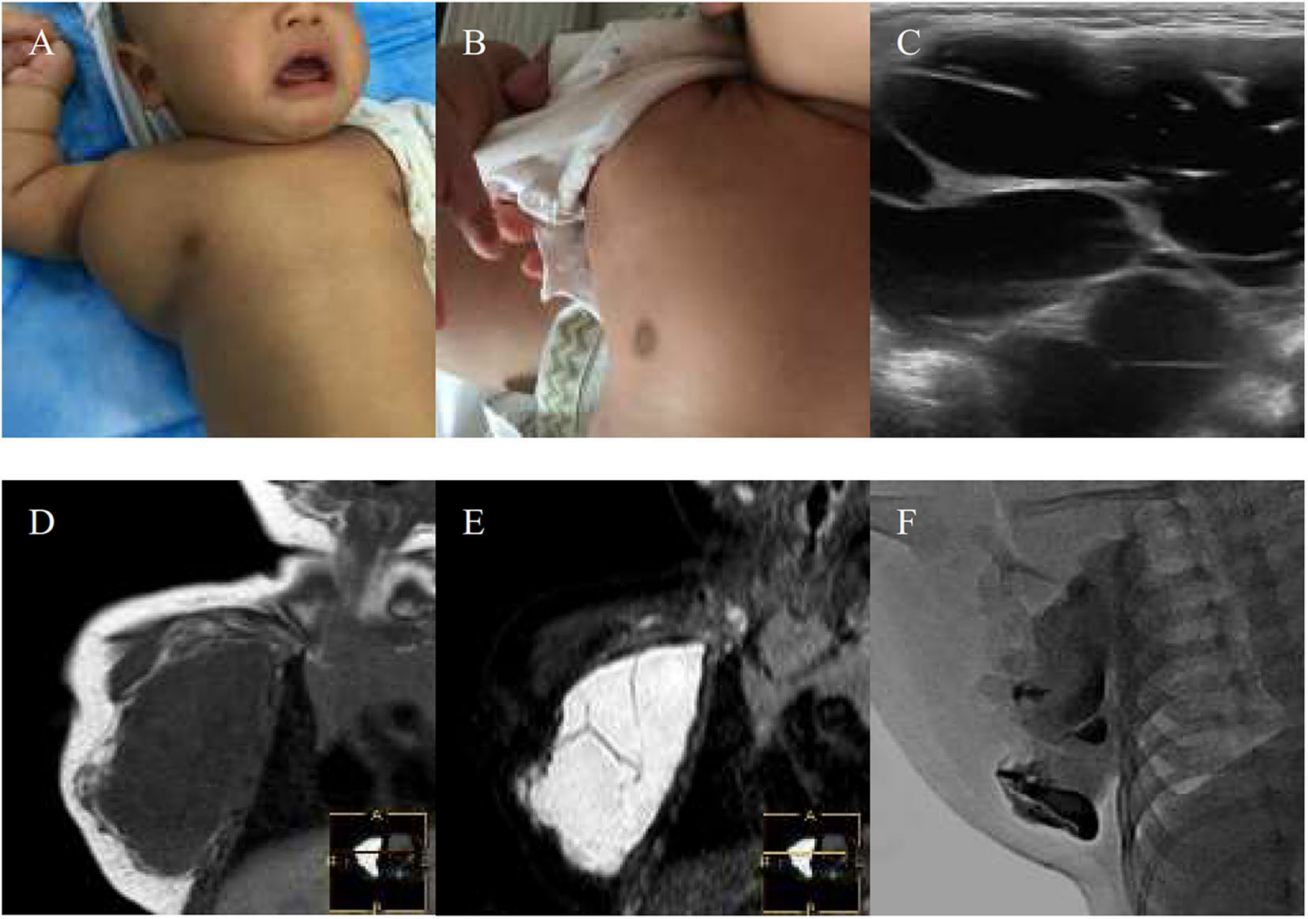
A 1-month-old boy with macrocystic lymphatic malformation (LMs) located in the right subaxillary gets excellent resolution **(A–F)**. **(A)** Before treatment. **(B)** After bleomycin injections after one injection. **(C)** USG. Bleomycin-visipaque mixture filled the majority of the cystic **(F)** compared with pre-MR **(D,E)**.

**Figure 2:**
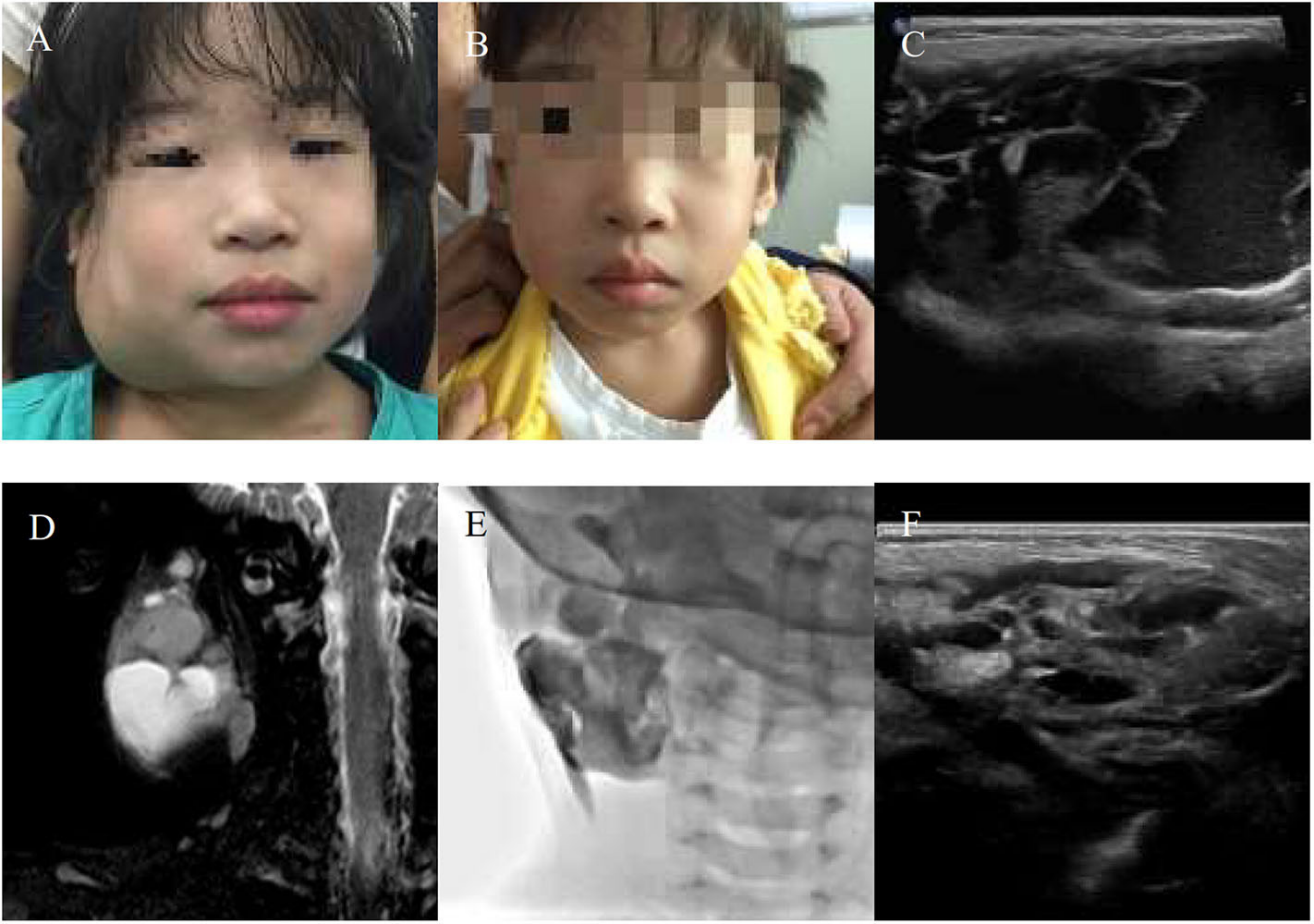
A 10-year-girl with combined lymphatic malformation (LMs) in the right parotid region gets excellent resolution **(A–F)**. **(A)** Before treatment. **(B)** After bleomycin injections after one injection. **(C,D)** Some radiological features are sufficiently suggestive of diagnosis of LMs. **(E)** The lymphatic malformation filled with bleomycin-visipaque mixture. **(F)** Reexamin ultrasonography shown a few of the residual lesions.

**Figure 3:**
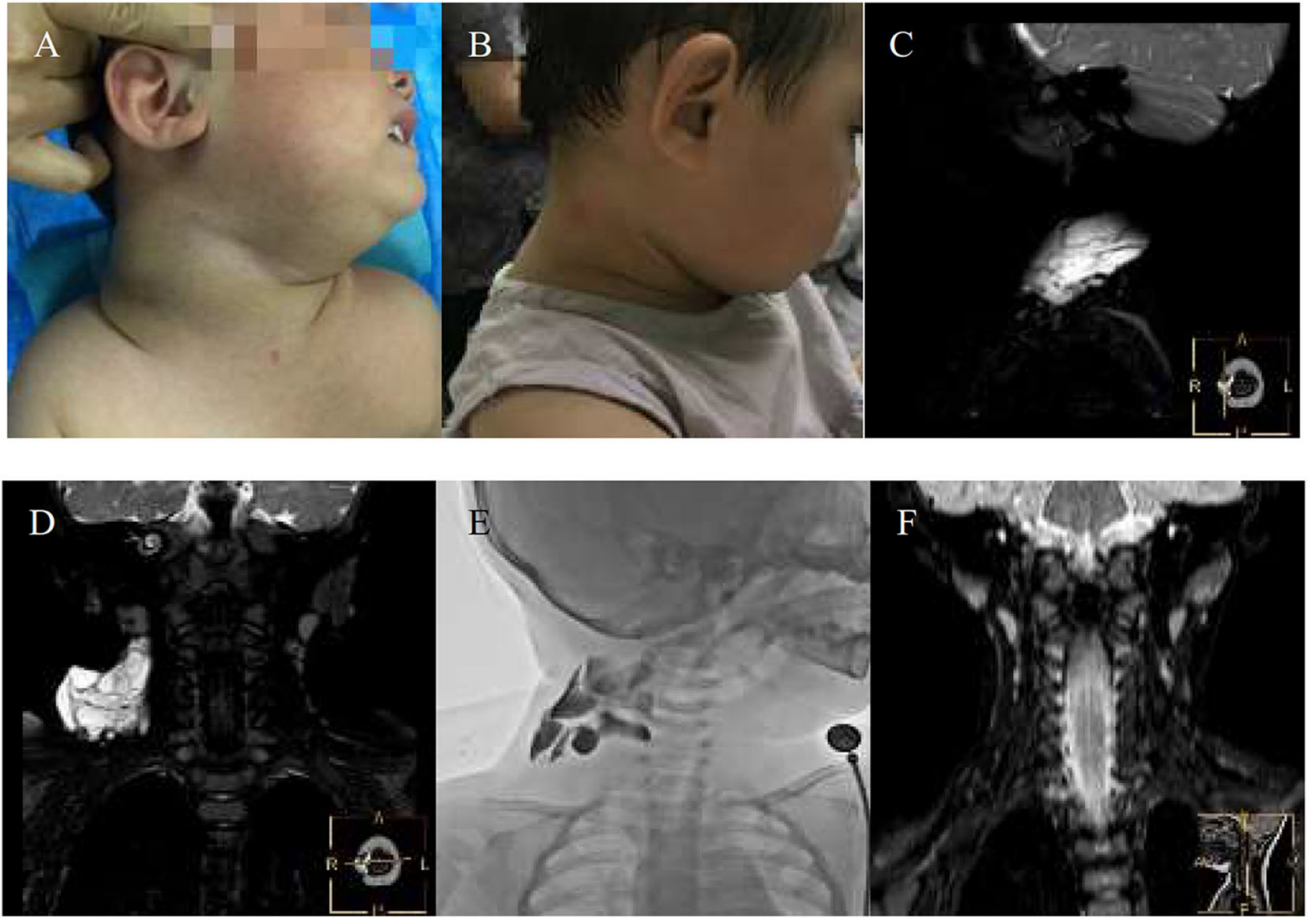
An 11-month-girl with combined lymphatic malformation (LMs) in the right maxillofacial region gets excellent resolution **(A–F)**. **(A)** Before treatment. **(B)** After bleomycin injections after two injections. **(C,D)** The sagittal T2 and coronal T2 demonstrate a cervical cyst. **(E)** The lymphatic malformation filled with bleomycin-visipaque mixture. **(F)** There is no recurrence at following-up 9 months.

**Figure 4:**
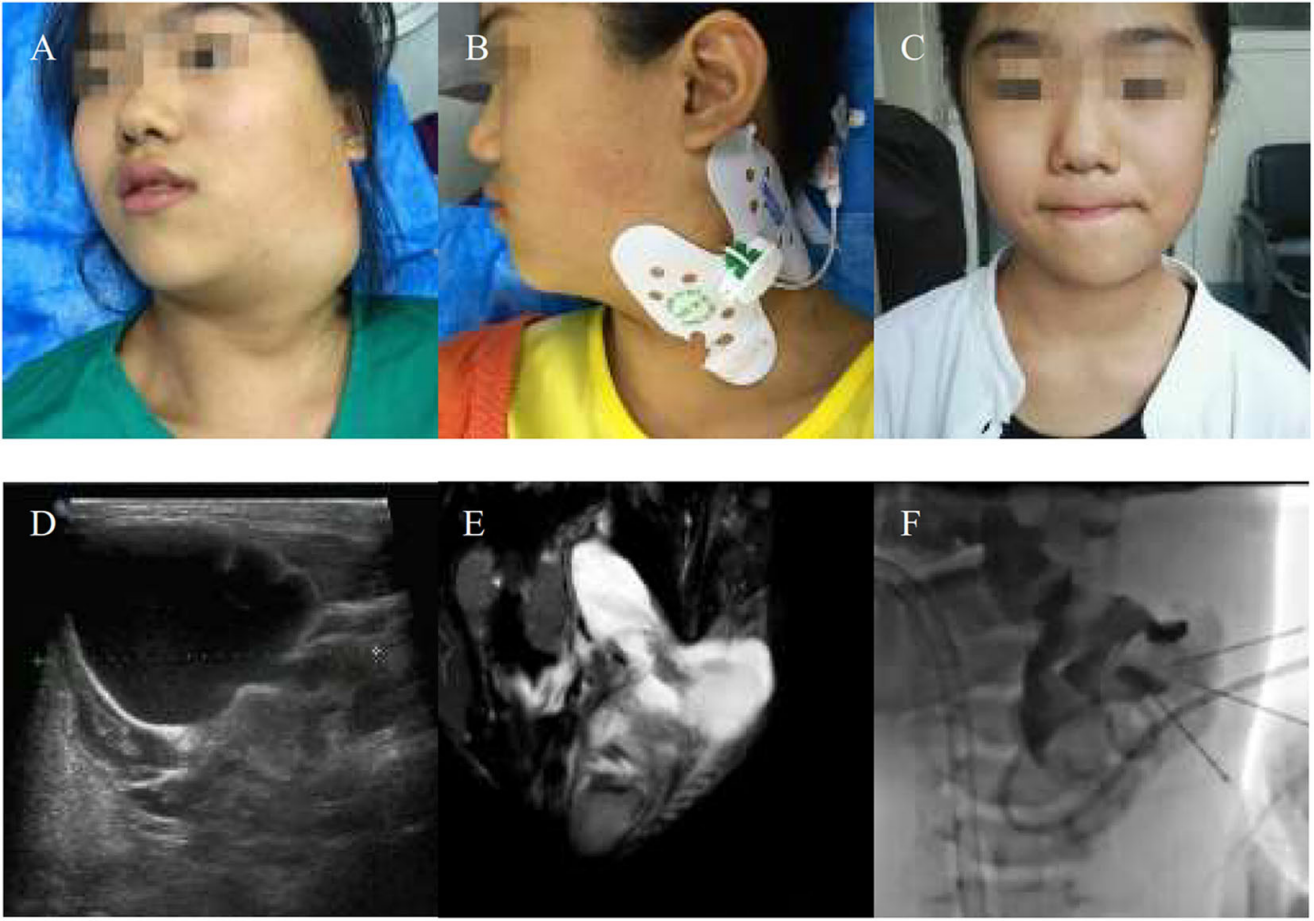
An 11-years-girl with combined lymphatic malformation gets excellent resolution. **(A)** The left neck mass with pain and dysfunction increased rapidly. **(B)** The bleomycin- visipaque mixture was injected through drainage tube. **(C)** An excellent resolution was got at follow-up 2 months. **(D)** Findings are consistent with a primarily macrocystic lymphatic malformation with a small amount of microcystic components. **(F)** The lymphatic malformation filled with bleomycin-visipaque mixture **(E)**.

The number of treatment session per patient varied from 1 to 5, with a mean of 1.34 sessions. The average number of treatment sessions for large cystic LMs were 1.26, and the effective of rate of excellent resolution or significant improvement was 95.9% (*n* = 69) and 4.1% (*n* = 3). The average frequency of treatment of microcystic lymphangioma were 1.45 sessions, and the effective rate was 50% (excellent resolution, *n* = 9), 27.8% (significant improvement, *n* = 5), 16.7% (moderate, *n* = 3) and 5.5% (mild, *n* = 1). The average frequency of treatment of mixed LMs were 1.41 sessions, and the effective rate was 63.9% (excellent resolution, *n* = 39), 29.5% (significant improvement, *n* = 18), 4.9% (moderate, *n* = 3) and 1.6% (mild, *n* = 1). The results showed a statistically significant therapeutic effect between every type of LM (*P* < 0.05). In total, excellent visual and radiological resolution was observed in 77% (117/151) of lesions (Figures 1–4), 17% (26/151) exhibited significant improvement, and 8 patients experienced a slight response. Unfortunately, more treatment and worse results were obtained in those with microcystic tumors. For 18 patients, four with poor results, one lost to follow-up, and six lesions received repeated treatment.

A total of 151 patients underwent interventional operation in a total of 203 sessions, in which 113 (74.8%) required single dose, 26 (17.2%) required two doses, 11 (7.3%) required three doses and 1 (0.7%) required five doses of intralesional bleomycin. No serious side effects were recorded according to the Society of Interventional Radiology criteria classification system for complications (10). Out of the 72 patients with large cystic LMs, 58 (80.6%) required single dose, 11 (15.3%) required two doses, 2 (2.8%) required three doses, and 1 (1.4%) required five doses of intralesional bleomycin. Out of the 18 patients with micro-cystic LMs, 12 (66.7%) required single dose, 4 (22.2%) required two doses, and 2 (11.1%) required three doses of intralesional bleomycin. Out of the 61 patients with mix-cystic LMs, 43 (70.5%) required single dose, 11 (18%) required two doses, and 7 (11.5%) required three doses of intralesional bleomycin. However, minor side effects included skin erythema at the injection site, local swelling, mild tenderness and fever, which were controlled by oral antipyretics. No patient developed excessive scarring as a result of the procedure(s).

## Discussion

Treatment of LMs using conventional percutaneous sclerotherapy techniques has been widely applied. Despite some of the advances made over time (6, 11), a more effective, feasible, and minimally invasive treatment is urgently required. In our experience, treatment may have to be repeated with the use of US guidance alone (7), which is sometimes unable to distinguish the sclerosing agent from the original cyst fluid, thus leading to missed cysts. We introduce a method of simulated angiography using a bleomycin mixture based on single-frame DSA fluoroscopy and US guidance that provided encouraging results in a fewer number of treatment sessions. Improve this process and shorten hospital stay.

Sclerotherapy provides a treatment option in such patients, offering a viable alternative to surgery, especially in those with macrocystic LMs (12, 13). The sclerosant agent damages the epithelial lining of cystic spaces, with a subsequent decrease in lymph fluid production and collapse of the cysts. Various agents, such as bleomycin, OK-432, alcoholic solution and doxycycline, have been used for percutaneous sclerotherapy. Pradyumna et al. (14) reported complete resolution in 16 (44.4%), good in 17 (47.2%), poor in 3 (8.3%) patients, and repeated treatment in 33 (92%) patients treated with bleomycin. In a study including 17 cases of lymphangioma, Erikçi et al. (15) reported good response in 50% of the lesions, complete resolution in 35.7%, and poor response in 14.3%. OK-432 was proposed as a safe and effective treatment with a long lasting effect in the management of macrocystic LMs (16). Ogita proposed OK-432 as a treatment for LM in 1987. Kim DW et al. (17) found that response to treatment was complete or good in 13/20 macrocystic lesions and in 0/6 microcystic lesions performed using OK-432. Thus, a significant relationship was observed between the type of lymphatic malformation and the success of OK-432 sclerotherapy (*P* = 0.0149).

Doxycycline is an inexpensive and widely available antibiotic that induces dense adhesions and fibrosis within target vascular malformations (18). A previous study reported that 87% of patients (28 of 32) exhibited excellent or moderate response with an average of 2.8 treatments (range, 1–7 treatments) with doxycycline (19). However, this agent can cause serious side effects, such as hemolytic anemia, hypoglycemic and metabolic acidosis, and transient hypotension (20). In our center, we have favored bleomycin, considering that the majority of our patients are < 1 year of age. Previous investigations have revealed that there is no consensus regarding the type of sclerosant such as picibanil (OK-432) and bleomycin. A prospective study showed that out of five patients of mixed variety, two (40%) showed good response and three (60%) showed a poor response and more frequency of treatment has been reported (14, 16). The reason for this is that it is not intuitive to determine the distribution of drugs usually through image guidance performed with only US. The desired effect of the sclerosant is associated with the drug per unit surface area of the lesion. More importantly, US can be limited in evaluating drug distribution in lesion, so fluoroscopy and MR images fusion used in evaluation would be more precise (21). This may be one reason for some poor results and the need for repeated treatment. Visualization of LMs using US can be challenging, particularly when these lesions are located deep in the head and neck space, or be obscured by adjacent bony structures. We provide a combination regimen of percutaneous sclerotherapy using US to reduce radiation exposure, such as real-time fluoroscopy-guided treatment, which is a key consideration in children, and single-frame fluoroscopy using DSA compared with the pre-MRI to estimate an adequate therapeutic window. We believe this is important for an immediate good result.

Safe and accurate percutaneous delivery of sclerosant into the LM without extravasation into the surrounding soft tissue or flow into the draining veins is important to limit treatment complications (21). The patients in our study had to remain in the hospital for at least 3 days and were followed up in the outpatient clinic for timely detection of complications. We are trying to improve this process and shorten hospital stay. No serious complications, such as skin necrosis, occurred, which may be related to the lack of intraoperative extravasation.

In conclusion, obtaining good visualization of the interface and fewer injections are other challenges on treating LM, and new approaches to the management of these lesions must be developed. Simulated angiography using a bleomycin mixture for LMs in children was feasible based on our institutional experience. Nevertheless, a prospective randomized trial should compare conventional treatment with this promising new technique.

## Data Availability Statement

All datasets presented in this study are included in the article/supplementary material.

## Ethics Statement

The studies involving human participants were reviewed and approved by Qilu children's hospital of Shandong university ethics committee. Written informed consent to participate in this study was provided by the participants' legal guardian/next of kin. Written informed consent was obtained from the individuals and minors' legal guardian for the publication of any potentially identifiable images or data included in this article.

## Author Contributions

XL conceived and designed the study. LG and CW were responsible for literature review and article writing. DS and CW were responsible for the revision and editing of the manuscript, while JS and YZ analyzed the data. All authors reviewed the manuscript and approved the submitted version.

## Conflict of Interest

The authors declare that the research was conducted in the absence of any commercial or financial relationships that could be construed as a potential conflict of interest.
